# Wintering North Pacific black-legged kittiwakes balance spatial flexibility and consistency

**DOI:** 10.1186/s40462-015-0059-0

**Published:** 2015-10-21

**Authors:** Rachael A. Orben, Rosana Paredes, Daniel D. Roby, David B. Irons, Scott A. Shaffer

**Affiliations:** Department of Ocean Sciences, Long Marine Lab, University of California Santa Cruz, Santa Cruz, CA 95060 USA; Department of Fisheries and Wildlife, Oregon State University, Hatfield Marine Science Center, 2030 SE Marine Science Dr., Newport, OR 97365 USA; Department of Fisheries and Wildlife, 104 Nash Hall, Oregon State University, Corvallis, OR 97331-3803 USA; US Geological Survey-Oregon Cooperative Fish and Wildlife Research Unit, Oregon State University, 104 Nash Hall, Corvallis, OR 97331-3803 USA; U.S. Fish and Wildlife Service, 1011 East Tudor Road, MS 341, Anchorage, AK 99503 USA; Department of Biological Sciences, San Jose State University, One Washington Square, San Jose, CA 95192-0100 USA; Institute of Marine Sciences, Long Marine Lab, University of California Santa Cruz, California, 95060 USA

**Keywords:** Black-legged kittiwake, *Rissa tridactyla*, Non-breeding, Geolocation, Bering Sea, Colony, Sex, Fidelity, Oceanographic habitats

## Abstract

**Background:**

Marine environments are inherently dynamic, yet marine predators are often long-lived and employ strategies where consistency, individual specialization, routine migrations, and spatial memory are key components to their foraging and life-history strategies. Intrinsic determinates of animal movements are linked to physiological and life-history traits (e.g. sex, colony, experience), while extrinsic influences occur as the result of an animal’s interactions with either other animals or the environment (e.g. prey availability, weather, competition). Knowledge of the factors affecting animal movements is critical to understand energetic bottlenecks and population dynamics. Here, we attempt to understand the interaction of some of these factors on the winter distributions of a surface-feeding seabird in the North Pacific. Between 2008 and 2011, we tracked 99 black-legged kittiwakes breeding at St. Paul and St. George in the Pribilof Islands, Alaska using geolocation loggers. We tested for colony and sex differences in winter distributions, and individual spatial fidelity over two consecutive winters of 17 individuals. Then we linked tracking data to associated environmental conditions as proxies of prey availability (e.g. sea surface temperature, mesoscale eddies, chlorophyll a, and wind) to understand their influence on kittiwake space use at an ocean basin scale.

**Results:**

Black-legged kittiwakes from both Pribilof Islands primarily wintered in pelagic sub-arctic waters, however, distributions spanned seven ecoregions of the North Pacific. There was a high degree of similarity in area use of birds from the two closely situated colonies and between sexes. Birds tracked for two consecutive years showed higher fidelity to wintering areas than occurred at random. Annual changes were apparent, as distributions were further north in 2009/10 than 2008/09 or 2010/11. This occurred because 70 % of birds remained in the Bering Sea in the fall of 2009, which corresponded with lower October sea surface temperatures than the other two years.

**Conclusions:**

Although individuals returned to wintering areas in consecutive years, our results suggest that under current conditions individual black-legged kittiwakes have a high capacity to alter winter distributions.

## Background

Marine environments are inherently dynamic, yet marine predators are often long-lived and employ strategies where consistency, individual specialization, routine migrations, and spatial memory are key components to their foraging and life-history strategies [1–3]. At small scales, prey species such as squid, fish, and krill are patchily distributed—both vertically and horizontally in the water column [4, 5]. Surface foraging seabirds are adapted to find food at small scales through social communication, olfactory and visual cues [6, 7]. During the non-breeding period many of these species travel thousands of kilometers to upwelling regions and frontal zones where prey are more predictable [8, 9]. This results in individuals choosing migratory destinations at an ocean basin scale, presumably without knowledge of conditions at their destinations [10].

Where and how animals move across a landscape is driven by a myriad of factors than can be simplified into intrinsic or extrinsic factors [11, 12]. Intrinsic determinates of animal movements are linked to physiological and life-history traits of species (e.g. reproductive status, past experience, physiological needs and capacity, navigation abilities), while extrinsic influences occur as the result of an animal’s interactions with either other animals or the environment (e.g. competition, predation, prey availability). While disentangling intrinsic and extrinsic factors and understanding how they may interact is difficult [13], intrinsic factors appear to be particularly important. For example, for seabirds, age, sex, and breeding colony may influence migrations [14–18]. As a consequence, these differences imply individuals within populations will be differentially affected by extrinsic factors such as prey availability. Furthermore, there is undoubtedly an influence of past experience and learning that shapes some portion of migratory paths, wintering areas, and prey preferences in long-lived seabirds [19–24]. Yet, over a lifetime individuals are likely to be flexible in their foraging choices as oceanic habitats are dynamic and conditions change from one season to the next [25, 26]. Understanding how intrinsic and extrinsic factors interact to influence animal movements is paramount for an understanding of population trends and spatial capacity, particularly in highly mobile long-lived species.

Black-legged kittiwakes show spatial and dietary variability in foraging habitats both within and between colonies [27, 28], and throughout the annual cycle, including the winter non-breeding period [29–33]. Breeding individuals are known to display marked fidelity to locations and foraging in concert with tidal cycles [34], yet little is known about the importance of fidelity during the non-breeding period (but see [35]). In this study, we use the black-legged kittiwake as a model species to understand the importance of flexibility and consistency during their winter migrations to the dynamic pelagic environment. First we address the effects of breeding colony location, sex, and individual experience on the winter distributions of the black-legged kittiwake breeding at the Pribilof Islands. Then, we link three winters of kittiwake tracking data to associated environmental conditions and oceanic habitats to understand how annual habitat conditions influence space use at an ocean basin scale.

## Methods

To study black-legged kittiwake (hereafter kittiwake) wintering ecology, geolocation loggers (1.8/2.5/2.5 g, Mk13/Mk9/Mk19, British Antarctic Survey, Cambridge UK) were deployed on 157 kittiwakes during July of 2008–2010 at two colonies in the Pribilof Islands, Alaska (St. Paul Island, 57.17N 169.60W and St. George Island, 56.60N 169.60W; Table 1). All birds were captured off active nests using either a noose pole or foot snare supplemented with a CO_2_ powered net gun containing custom nets (Super Talon Animal Catcher, Advanced Weapons Technology, California) at recapture. At deployment and recapture birds were weighed and measured, nest contents were recorded and blood samples for sexing via DNA were taken [36]. Overall, 86.6 % of birds were resighted and 77 % of loggers were recovered (Table 1). Seven loggers failed during data recovery and 13 loggers failed before recapture. Over the three study winters, 17 birds carried loggers for 2 winters. A total of 113 complete winter trips were recorded from 99 kittiwakes. It is possible that though the loggers used in this study were small (~0.6 % body mass or less) they still may have had deleterious effects on the individuals carrying them [37, 38], however no negative effects have been thus far reported for kittiwakes carrying geolocation loggers in terms of body mass, breeding participation or survival [29, 31, 32].

**Table 1:** Sample sizes of black-legged kittiwakes (*Rissa tridactyla*) on which geolocation loggers (GLS) were deployed, resighted and recaptured

	2008	2009	2010	Total
St Paul				
deployed	27^a^	24^b,c^	31	82
resighted	23 (85 %)	19 (79 %)	27 (87 %)	69 (84 %)
recaught	22 (81 %)	18 (75 %)	19 (61 %)	59 (72 %)
St George				
deployed	30^e^	17^f^	28	75
resighted	26 (87 %)	15 (88 %)	26^f^(92 %)	67 (89 %)
recaught	22 (73 %)	15 (88 %)	25 (89 %)	62 (83 %)

Birds were not individually marked in 2008 so resights rely on birds attending the same nests in subsequent years. Like recaptures, resights presented are cumulative, so birds from 2008 had three seasons of following effort, while birds from 2010 only had one year of recapture and resighting effort. Our objective was to recatch rather than resight birds, which often required hiding from birds rather than reading alphanumeric bands. Most birds were deployed with GPS and GLS loggers for a summer foraging study [27, 28], were subsequently recaptured and GLS loggers redeployed for overwinter. Thus deployment numbers also include 7 birds that were not recaptured during the summer breeding season (GPS tags fell off when tail feathers were molted); subsequently only 2 of these birds were recaught or resighted. Omitting these birds (and those whose nesting ledges fell) gives an overall resight of 91 %

^a^ Includes 1 bird deployed for the summer with a GPS and a GLS logger; the GPS was not recovered and the bird was not resighted in subsequent seasons

^b^ 3 birds were deployed in on a cliff face that collapsed overwinter and never resighted. These birds may have relocated to other areas of the colony where, due to the size of the cliffs, we were unable to resight them

^c^ Includes 2 birds deployed for the summer with a GPS and a GLS logger; the GPS was not recovered and birds were not resighted in subsequent seasons

^d^ Includes 2 birds deployed with both a GPS and GLS logger; the GPS was not recovered. 1 bird was recaught with a GLS logger in 2009, the other was not resighted

^e^ Includes 2 birds deployed with both a GPS and GLS logger; the GPS was not recovered. 1 bird was recaught with a GLS logger in 2010, the other was not resighted

^f^ High Bluffs, where 2 GLS loggers were deployed in 2010, was only visited twice in 2011 and 1 bird was never seen

Data processing, spatial analyses and statistical tests were conducted using MATLAB (v2014a, The Mathworks, Natick, MA) and R v3.1.1 (R Development Core Team, 2014). Significance was set to *p* < 0.05.

### Geolocation processing

Geolocations were estimated from September 1 thru May 30 with the colony as a fixed start and end location using the ‘TripEstimation’ package in R to implement a Bayesian model that incorporates a land mask and flight speed into the prior distribution and calculates locations from light levels using the ‘template-fit’ method [39–41]. We used a mean speed of 33 km hr^−1^ [42], however tag derived activity data (time spent dry, [32]) indicated that birds only spent 15 ± 9 % of each 24 hr period flying, therefore, as a conservative estimate we chose to assume that birds spent 33 % (two standard deviations from the mean) of their time in flight, restricting an individual’s range over 12 hours, on average, to 130km. Thus a mean speed of 10.89 km hr^−1^ and speed variance equal to half this were entered into the model to parameterize a log-normal distribution. The most probable track was then obtained through a Kalman filter with six Markov Chain Monte Carlo (MCMC) simulations of 1000 iterations each, after a 500-iteration burn in period. Tracks were visually compared and found to be similar to those calculated using smoothing and filtering methods that result in errors of 186 ± 114 km [43]. Subsequently, all analysis were constrained to October thru February to avoid greater errors inherent in locations estimated around the equinox period.

### Distributions

How birds share space over large scales relates to the size of individual ranges, the distribution of these ranges, and the density of birds. Individual range size was calculated as the number of 45 × 45 km grid squares occupied by the last chain of the MCMC estimation (1000 iterative tracks). To assess how variable each group (colony, sex, year) was in the areas they used as well as a quantitative assessment of sample sizes, the cumulative number of grid cells occupied was calculated with the addition of one track from each individual randomly selected for 10,000 iterations [44]. Groups with less variability or higher amounts of area shared between individuals, have shallower curves than groups with high individual variability in area use [44]. To assess the amount of shared area, an index of similarity was calculated as the ratio of the number of shared grid cells (45 km) occupied by the last chain of the MCMC estimation to the total grid cells used by both groups, where identical groups would be equal to 1. To test these indexes against what similarity might randomly occur, birds were randomly assigned to groups and the index of similarity was calculated 10,000 times with the same number of individuals in each group as the original dataset. *P*-values were calculated as the percentage of iterations that resulted in an similarity index smaller than observed [32, 45]. Finally, to assess relative density of birds and overall distributions, densities were calculated from the last chain of the MCMC estimation for each individual over a 5 × 5 km grid (to aid in visualization). This method incorporates the uncertainty in the location estimates and negates the reasons to use a method such as a kernel density estimate [40, 46].

### Individual Spatial Fidelity

Area fidelity can occur at varying spatial scales. To identify the spatial scales in which kittiwakes showed fidelity to wintering areas we calculated a monthly index of similarity, a measure of the number of shared grid cells, between all estimated locations for repeat trips from individual birds (*n* = 17) at a series of grid sizes ranging from 10–400 km. Then to quantify a random measure of overlap we calculated the percentage of overlap between 59 random pairs, between individuals of the same sex and colony from consecutive years.

### Annual difference in large marine ecosystem use

To better understand annual changes in large-scale distributions and habitat use of kittiwakes we assessed the use of oceanic biogeographical provinces of the North Pacific [9]. For each province, at the scale of 45 km grid cells, we calculated the percentage of the biogeographic province occupied by kittiwakes (again incorporating location error by using the last chain of the MCMC estimates) (# grid cells occupied/total grid cells in province), average density of occupied grid cells, and the proportion of the overall bird distribution in each province (# grid cells occupied inside region/total # grid cells occupied). We also assessed how individuals used these regions by calculating the number of ecoregions used by individual birds and by sexes.

### Annual habitat conditions

Habitat variables were extracted along the best-fit tracks at a 1 ° grid scale. Sea surface temperatures (SST) were extracted as an eight-day blended product from data hosted by NOAA’s Environmental Research Division (http://oceanwatch.pfeg.noaa.gov/thredds/catalog.html). Bathymetry was extracted from the ETOPO1 dataset [47], and bathymetric slope calculated. As eddies are known to condense prey for surface foraging predators including kittiwakes [28, 48], we extracted sea surface height (SSH) and surface currents used to calculate eddy kinetic energy (EKE) were extracted from the Navy Layered Ocean Model (1/32 °, http://www7320.nrlssc.navy.mil/global_nlom/) using the nctoolbox (https://github.com/nctoolbox). We calculated the distance to eddy edge using mesoscale eddy trajectories [49]. Distance to productive seamounts or knolls, defined as those within 1500m of the sea surface, was calculated for each bird location, as these features are known to enhance biological productivity [50, 51]. Monthly composites of MODIS-Aqua chlorophyll a, spanning 2007–2012, were constructed using DINEOF 3.0 to interpolate regions where clouds obscured satellite data [52]. Locations were matched to monthly composites, with locations from the first week of each month assigned to the previous month. Wind speed was extracted from the RNCEP Reanalysis II data sets for surface values [53, 54].

#### Residency time

We used residency time to understand the influence of habitat variability on individual movements. We chose this parameter rather than using a metric of foraging based on wet-dry data [32], as kittiwakes can employ both active and sit-and-wait foraging styles [55]. Residency time can be defined as the cumulative amount of time an individual animal spends within a circle of constant radius over a period of time [56]. In our case, we chose a radius of 45 km, as this is rougly equivelant to the mean daily distance kittiwakes traveled over the whole non-breeding period (September–May), and a time constraint of 1 month to avoid false positives if individuals crossed over their own path months later on the return trip. High residency locations, indicating periods of intense search effort, were then chosen as the upper quartile of each individual’s residency times [57].

#### Habitat selection models

Linear-mixed models were used to relate residency time to oceanic habitat characteristics in the top four biogeographic regions frequented by kittiwakes. Variance inflation factors were calculated, but all were <3, so all explanatory variables were retained [58]. To meet the conditions of normality for linear models residency time and chlorophyll a were log transformed and distance to eddy and bathymetric slope were square-root transformed. Best-fit models for each ecosystem were constructed using a reductive approach and identified from Akaike Information Criterion (AIC) scores based on restricted maximum likelihood estimates [58]. Individual was included as a random effect. Marginal R^2^ values were used to describe the variance explained by the fixed effects and conditional R^2^ values, the combined fixed and random effects [59, 60].

## Results

**Fig. 1:**
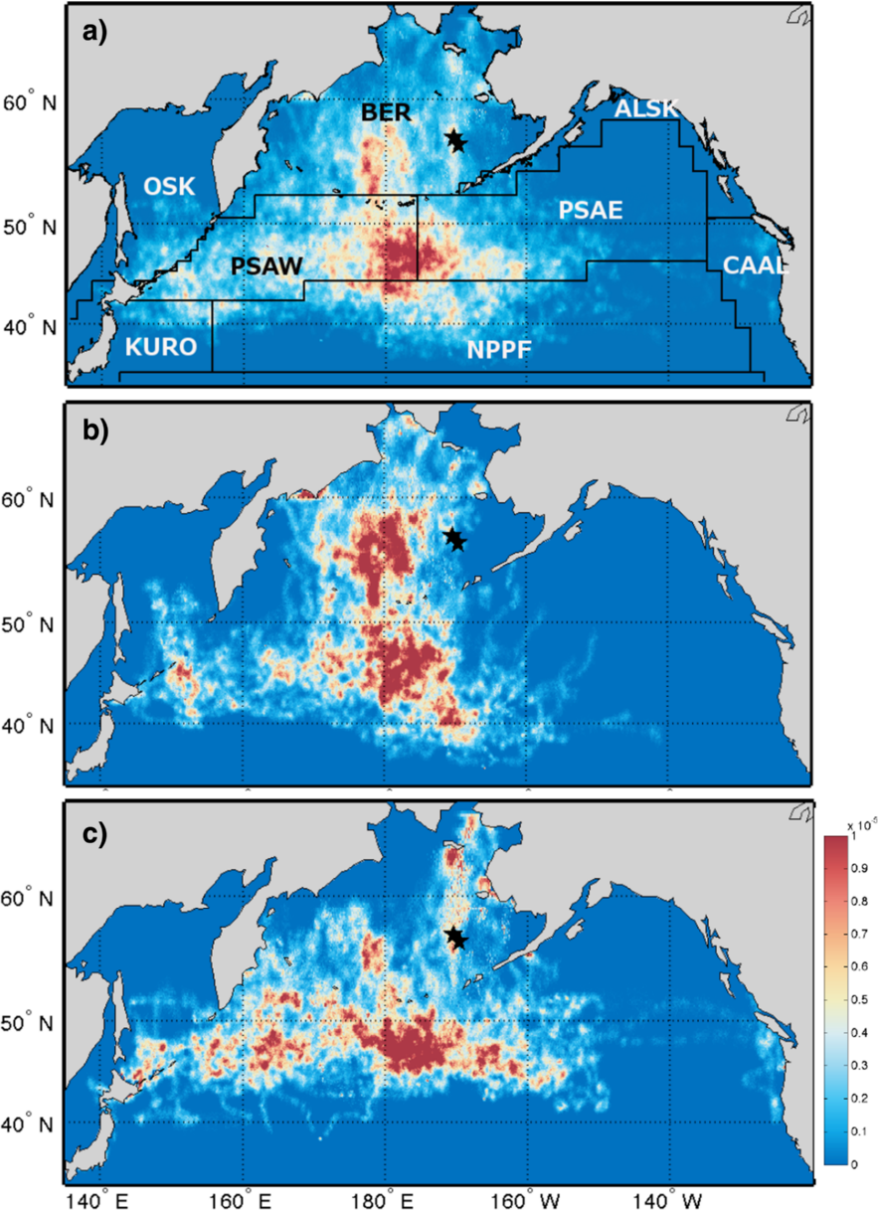
Annual distribution of black-legged kittiwakes (*Rissa tridactyla*) from the Pribilof Islands during their central wintering period (October thru February). In **a** 2008/09 (*n* = 38), **b** 2009/10 (*n* = 44), and **c** 2010/11 (*n* = 33). The boundaries of the ecoregions are shown following Longhurst, 2010, with the Bering Sea [BER] and Sea of Oskhosk [OSK] separated into two subregions. Remaining abbreviations are as follows: ALSK = Alaska Coastal Downwelling Zone, CAAL = California Current, KURO = Kuroshio Current, NPPF = North Pacific Polar Front, PSAE = Eastern Subarctic Gyre, PSAW = Western Subarctic Gyre

**Fig. 2:**
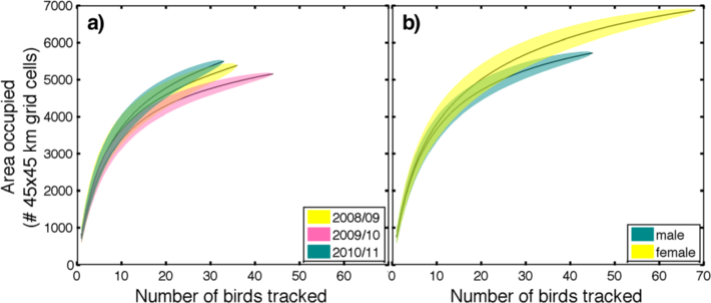
Area occupied (number of 45km^2^ grid cells) by migrating black-legged kittiwakes (*Rissa tridactyla*) from October thru February. By **a**) year where 2008/09 = *yellow*, 2009/10 = *pink*, and 2010/11 = *green* and **b**) sex (female = *yellow*, male = *green*)

**Fig. 3:**
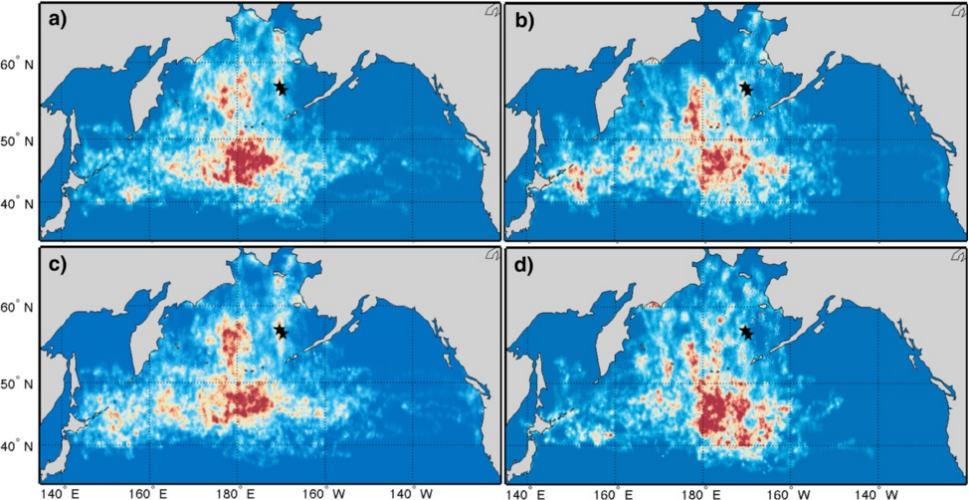
Distributions of black-legged kittiwakes (*Rissa tridactyla*) from the Pribilof Islands during their central wintering period (October thru February) for colonies and sexes. From **a** St. Paul (*n* = 60), **b** St. George (*n* = 53), **c** females (*n* = 76) and **d** males (*n* = 56)

**Fig. 4:**
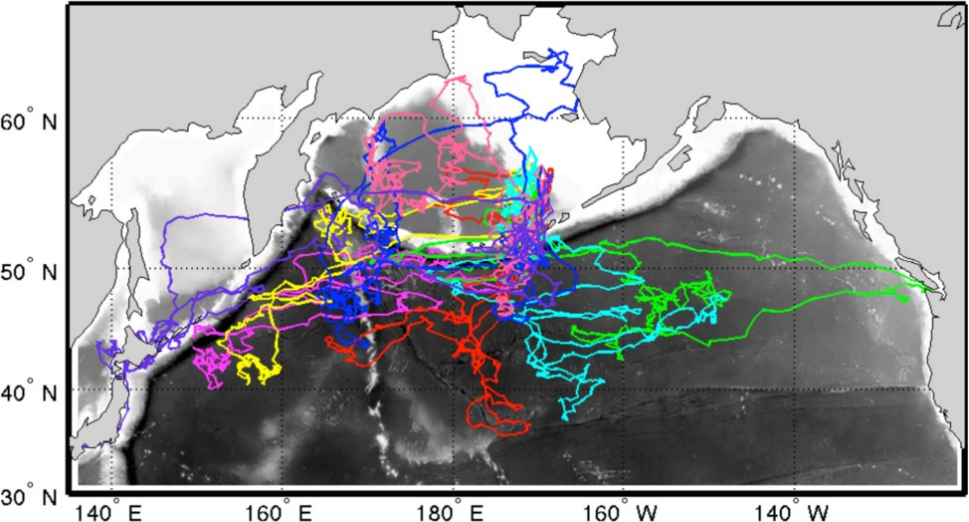
Variation in migratory route shown by 8 example tracks of black-legged kittiwakes (*Rissa tridactyla*) originating from the Pribilof Islands

Kittiwakes from the Pribilof Islands wintered predominantly across the deep oceanic waters of the central and western subarctic North Pacific all three years (Fig. 1). Area use of individual birds did not significantly differ between years, sexes, or colonies (1,557,000 ± 358,000 km^2^, *p* > 0.05; Table 2). As a group, females covered more area than males (Figs. 2 and 3). There was no difference in the size of cumulative area covered by birds from the two study colonies up to 40 individuals (*n* = 40, St Paul: 12,328,000, St George: 12,235,000), however at this point the lines diverge suggesting more low level variation in area use occurred among St. Paul birds (*n* = 53, St Paul: 13,241,000, St George: 12,235,000; Figs. 2 and 3). Only birds from St. Paul used areas of the northwestern Bering Sea (Fig. 3). The amount of overlap between groups was not different than random for colonies (72 % overlap, *p* = 0.113) and marginally different between sexes (70 % overlap, *p* = 0.06). Yearly combinations all showed less overlap between the observed distributions than randomly grouped tracks (2008/09 vs. 2009/10, 63 % overlap, *p* < 0.001; 2009/10 vs. 2010/11, 56 % overlap, *p* < 0.001; 2008/09 vs. 2010/11, 61 % overlap, *p* = 0.001). A small number of birds, all females (*n* = 5), traveled east to the California Current System. Overall, kittiwakes showed a wide variety in wintering routes (Fig. 4).

**Table 2:** Yearly summary of winter space use for black-legged kittiwakes (*Rissa tridactyla*) from the Pribilof Islands (October thru February, 2008–2011)

	2008/09	2009/10	2010/11
# of birds St Paul [male / female]	19 [7/12]	26 [12/14]	15 [3/12]
# of birds St George [male / female]	17 [7/10]	18 [10/8]	18 [6/12]
Daily distance traveled (km)*	35.4 ± 3.5	32.1 ± 2.3	34.5 ± 2.8
Max distance from colony (km)	2,573 ± 682	2,508 ± 625	2,601 ± 798
Individual Area use (# 45 km grid cells)	811 ± 226	726 ± 120	781 ± 172
Residency (days)	2.17 ± 0.35	2.45 ± 0.45	2.09 ± 0.37

*year: F = 14.25, *p* < 0.001

### Scale of Individual Spatial Fidelity

**Fig. 5:**
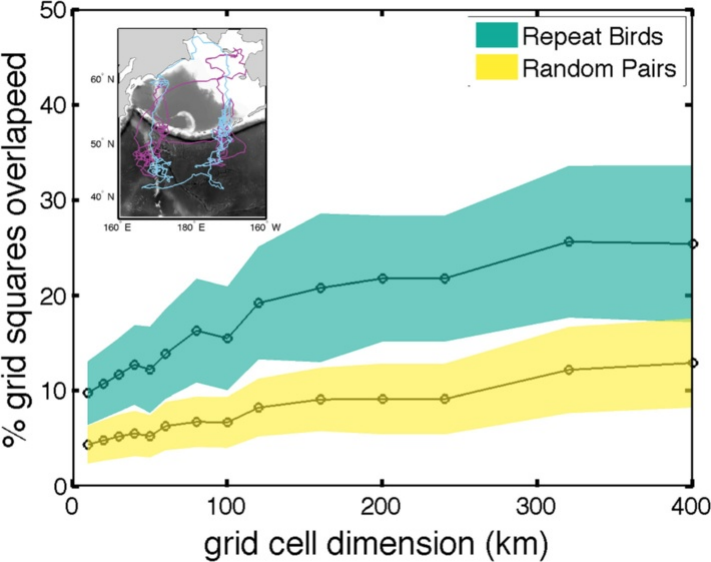
Higher site fidelity in repeat migrations of 17 black-legged kittiwakes (*Rissa tridactyla*) during October thru February than randomly paired tracks (*n* = 59), of the same colony and sex. Inset: example track from a kittiwake breeding at St Paul where *dark purple* is 2009/10 and *light blue* is 2010/11

Birds that were tracked for two winters showed a tendency to return to the same regions and, for example, had on average 9 % overlapping grid squares for 100 × 100 km grid squares. During the mid-winter period (October - February) birds showed site fidelity at all scales, as the amount of shared grid cells was higher for individual bird repeat trips than for randomly paired tracks (Fig. 3). Some individuals repeated very unique routes, for instance, one bird traveled to the northern Bering Sea in the fall and then foraged along the Emperor Sea Mounts before heading back to the vicinity of the Pribilof Islands (Fig. 5).

### Annual difference in large marine ecosystem use

**Fig. 6:**
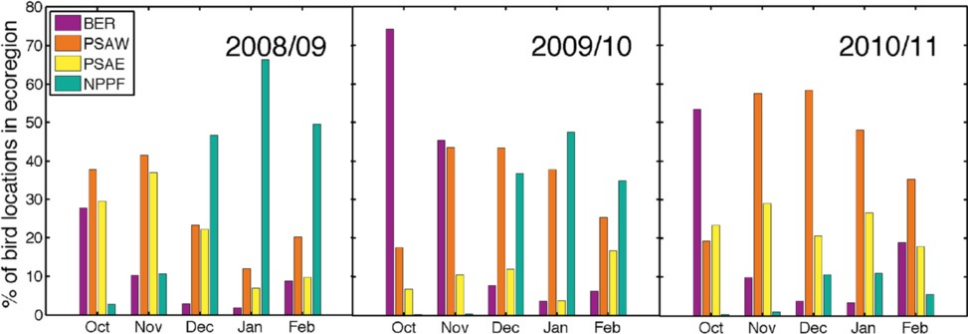
Percentage of monthly bird locations in the four North Pacific ecoregions frequented most by wintering black-legged kittiwakes (*Rissa tridactyla*) from the Pribilof Islands

**Fig. 7:**
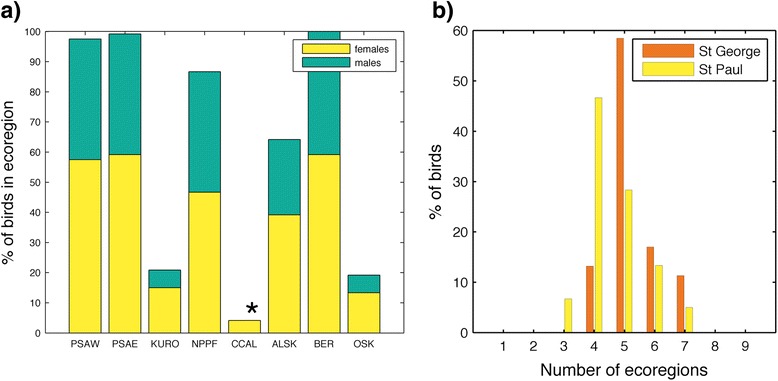
Ecoregion use of black-legged kittiwakes (*Rissa tridactyla*). **a** Percent of black-legged kittiwakes using each ecoregion, with the proportion of males in green and females in *yellow*. The California Current (CCAL), denoted by an asterisk, was visited exclusively by female kittiwakes. Counts of males and females in each ecoregion are not significantly different than the overall sample (Chi squared, *p* > 0.05). **b** Number of ecoregions used by individual birds from St Paul (*yellow*) and St George (*orange*)

Wintering Pribilof kittiwakes used seven biogeographical regions of the North Pacific (Table 3). On average 89.3 ± 4.4 % of the area kittiwakes used occurred in four of these, Epicontinental Seas (Bering Sea and Sea of Okhotsk—with little use of the Sea of Okhotsk), Western Subarctic Gyre (PSAW), Eastern Subarctic Gyre (PSAE), and the Polar Front (NPPF). Though geographically close to the Pribilof Islands, kittiwakes hardly used the Alaska Coastal Downwelling region (<3 % of bird distributions). In all three years, kittiwakes used almost the entire area of the PSAW, while distributions only occupied a portion of all other biogeographical regions (Table 3). The highest densities of tracked kittiwakes also occurred in the PSAW (Table 3). Annual differences were apparent in regards to when kittiwakes occupied each of the main ecoregions; specifically in October and November of 2009/10 there was a notable increase in the continued use of the Bering Sea (Fig. 3). The change occurred because 70 % of birds remained in the Bering Sea in the fall, which corresponded with lower October sea surface temperatures than the other two years (Table 4). In both 2008/09 and 2009/10, Dec–Feb, over 30% of bird locations were in the NPPF; while in 2010/11 the majority of locations during these months were farther north in the PSAW (Fig. 6). On average individual kittiwakes used 4.9 ± 1 ecoregions and this did not differ between sex, or year, however only female kittiwakes traveled to the California Current and birds from St Paul used fewer ecoregions (4.6 ± 1) than birds from St George (5.3 ± 0.08) (Fig. 7).

**Table 3:** Annual occupancy of marine biogeographical ecoregions by migrating black-legged kittiwakes (*Rissa tridactyla*) from the Pribilof Islands (October thru February)

	Average bird occupancy (# birds / 45km^2^)			% Ecoregion occupied			% bird distribution		
	2008/09	2009/10	2010/11	2008/09	2009/10	2010/11	2008/09	2009/10	2010/11
Epicontinental Seas:	3.9 ± 3.0	6.6 ± 5.3	4.9 ± 3.0	38.8	56.2	62.9	16.1	24.4	25.6
Bering Sea	4.3 ± 3.2	7.4 ± 5.5	5.6 ± 3.0	55.1	77.2	81.7	13.2	19.4	19.3
Sea of Okhotsk	1.9 ± 1.0	3.4 ± 2.2	2.6 ± 1.3	14.9	29.0	35.5	2.8	5.7	6.6
Subarctic Gyre (West)	8.5 ± 4.3	9.2 ± 5.2	7.6 ± 4.0	99.7	98.5	99.7	22.0	22.7	21.5
Subarctic Gyre (East)	6.6 ± 5.0	5.3 ± 5.6	5.4 ± 4.4	58.0	39.2	79.9	16.5	11.6	22.2
Alaska Coastal Downwelling	4.1 ± 2.4	1.4 ± 1.0	2.2 ± 1.4	20.5	12.1	34.5	1.4	0.8	2.2
Polar Front	4.5 ± 4.0	4.9 ± 4.2	2.3 ± 1.8	53.4	53.2	25.6	34.0	35.3	15.9
California Current	1.1 ± 0.3	0	1.5 ± 0.7	16.8	0	22.9	3.7	0	4.9
Kuroshio Current	3.3 ± 2.4	1.9 ± 1.1	1.1 ± 0.3	21.1	12.4	22.6	5.5	3.4	5.8

Marine biogeographical ecoregions are those defined by Longhurst [9]

**Table 4:** Habitat characteristics of wintering locations for black-legged kittiwakes (*Rissa tridactyla*) in the Bering Sea during October (2008, 2009, 2010)

	2008	2009	2010
Residency Time (d)	2.3 ± 1.0	2.9 ± 1.1	2.3 ± 1.3
SST (°C)	6.2 ± 0.8	5.8 ± 0.9	7.1 ± 1.4
Distance to Seamount (km)	474 ± 241	284 ± 144	438 ± 329
SSH (cm)	−7.9 ± 2.45	−7.3 ± 2.56	−9.6 ± 3.54
EKE (cm^2^ s^−2^)	66.8 ± 34.7	42.5 ± 27.1	48.3 ± 23.1
Distance to Eddy (km)	154 ± 62	139 ± 76	206 ± 121
Chl a (mg m^−3^)	1.61 ± 1.30	1.88 ± 2.63	3.0 ± 4.44
Wind speed (m s^−1^)	8.72 ± 1.33	8.71 ± 1.02	7.79 ± 0.99

Means ± SD are calculated from individual bird means

### Habitat conditions and selection

Throughout the winter, kittiwakes experienced a broad range of habitat conditions. Sea surface temperatures were on average 6.3 ± 2.6°C (daily individual range: −1.7–14.8 °C), chlorophyll a 1.32 ± 4.08 mg m^−3^ (0.25–62.67 mg m^−3^), EKE 63.9 ± 45.4 cm^2^ s^−2^ (5.8–437.2 cm^2^ s^−2^), SSH −8.9 ± 8.3 cm (−26.3–20.2 cm). While annual differences in habitat conditions existed, differences in habitat conditions between ecoregions were more pronounced (Table 3). Habitat variables were not a good predictor of residency in each ecoregion, with the best models only explaining <15% of the variation (Table 5).

**Table 5:** Summary statistics for linear mixed models of environmental influences on residency time for black-legged kittiwake (*Rissa tridactyla*) in each ecoregion

	df	AIC	ΔAIC	R^2^ (*m*)	R^2^ (*c*)
Bering Sea					
Full model	13	5504	7	0.005	0.149
d2ed + EKE + d2hill + wind	8	5497	-	0.004	0.150
Subarctic Gyre (West)					
Full model	13	13051	8	0.019	0.098
SST + d2ed + wind	7	13043	-	0.019	0.098
Subarctic Gyre (East)					
Full model	13	6129	10	0.028	0.071
SST + d2hill	6	6119	-	0.028	0.071
Polar Front					
Full model	13	8765	7	0.005	0.078
d2ed + d2hill + chla	7	8757	-	0.004	0.076

All models include a temporal correlation term (corCAR1(form = ~date|id)). Summary statistics of each full model (SST + depth + d2ed + slopetrn + EKE + ssh + d2hill + wind + chla) are presented first, followed by the best-fit model for each ecoregion. Akaike Information Criterion (AIC) were used to identify the best-fit model and marginal R^2^ (R^2^ (m)) and conditional R^2^ (R^2^ (c)) are presented. Abbreviations for the environmental variables used in the table are: sea-surface temperature (SST), distance to mesoscale eddy center (d2ed), sea-surface height (SSH), eddy kinetic energy (EKE), distance to productive seamounts and knolls (d2hill), monthly chlorophyll a (chla), bathymetric slope (slope) and bathymetry (bathy)

## Discussion

Kittiwakes from the Pribilof Islands underwent extensive pelagic migrations to diverse subarctic biogeographical regions in North Pacific. Both sex and past experience appeared to influence where individuals wintered. However, individuals displayed a large amount of flexibility and annual changes in distributions were larger than differences observed between sexes or colonies. As generalist predators kittiwakes are likely adapted to a high amount of environmental variability.

### Interplay of intrinsic influences

The reasons for colony specific wintering areas are not always clear. In some cases these colony differences may arise as the result of differing local conditions altering the phenology of migrations or simply be the result of a range expansion from geographically separated colonies as birds are still tied to these at an annual or biennial time scale [15, 30, 46, 61, 62]. Alternatively there are examples where birds from different colonies winter in the same region [61, 63]. The Pribilof colonies are only 70 km apart and the timing of breeding is highly synchronous [64]; thus it is not surprising that distributions of kittiwakes are similar. Yet, they are almost entirely distinct from wintering areas frequented by kittiwakes originating from Prince William Sound [33]. Therefore, there is likely additional colony associated variability in wintering areas across the North Pacific basin similar to that seen in kittiwakes in the North Atlantic [30].

We found a slight effect of sex on wintering distributions; though area use was largely similar, females appear to be more dispersive and no males traveled to the California Current System. Sex differences in distributions are often linked to differences in timing relative to nest defense or breeding roles and niche partitioning in sexually dimorphic seabirds [65–67]. Black-legged kittiwake males are larger than females in body and bill size [32] and this may lead to differences in energetic requirements or foraging preferences that may influence wintering distributions. Additionally, there is ample evidence of sexual differences in behavior of kittiwakes during the breeding season [27, 68, 69], and that breeding outcome (or elevated stress levels) may carry over to affect the non-breeding distributions of one but not the other sex [29, 70].

Fidelity during periods when individuals are constrained by central-place foraging, for instance in breeding seals and seabirds, appears to be relatively high [40, 71–73]. Much less is known about spatial fidelity when marine predators undergo migrations, often at the scale of ocean basins. Both flexibility and fidelity have been observed in migrating shearwaters, suggesting that individuals are able to explore and utilize multiple suitable wintering areas during a lifetime [24, 25]. Like migrating Atlantic puffins [20], black-legged kittiwakes displayed a tendency to return to areas that they frequented the year before. Both puffins and kittiwakes show characteristics of a dispersive migration, as routes often move gradually away from the breeding colony, rather than traveling to a distinct destination (see Fig. 4). However for kittiwakes, the amount of fidelity individuals showed was relatively small (~25 % of locations within 400 km grid squares) compared to the fidelity quantified for Atlantic puffins (median nearest neighbor distance <5 degrees) [20]. Kittiwakes are generalist predators that can only access the top 1 m of the water column, and they are much better fliers than puffins. Perhaps it is the combination of these life-history strategies that limits the amount of spatial fidelity that is advantageous, as prey resources can be patchy in pelagic environments [8]. Like puffins, it is unlikely that kittiwakes socially learn wintering areas as there is both individual fidelity and a high amount of variability within the population, instead it seems more likely that individuals rely on initial exploration, spatial memory and current conditions to inform travel paths. Our dataset is dominated by comparisons to 2009/10 when population level distributions were distinctly different from the other two years. This contrast likely led to less fidelity than might occur under similar conditions. More research is needed to understand if individuals are returning to areas where they previously experienced good foraging conditions and how fidelity changes when conditions change.

### Oceanographic habitats and annual conditions

During all three of our study years the highest densities of wintering kittiwakes occurred in the central subarctic Pacific at the confluence of the Western Subarctic Gyre (PSAW) and the Eastern Subarctic Gyre (PSAE). This region, characterized by the subarctic current may support relatively high zooplankton biomass in the summer [74, 75]. Kittiwakes from the Pribilofs used the entire PSAW. The PSAW has higher primary productivity than the Gulf of Alaska [76], more intense eddy activity [77], and supports a greater diversity of myctophid species [78]; potentially providing a more predictable winter habitat than the other ecoregions. Annual changes in kittiwake distributions occurred at a much higher magnitude, measured by the amount of overlap, than differences between intrinsic groups (colony and sex), and this reflects the contrasting conditions that occurred during the three-study years. Even restricted to ecoregions our models relating residency time and environmental variables were not able to explain much of this relationship. It may be, that as generalists, kittiwakes are foraging on a diverse suite of prey (with variable preferences for oceanographic conditions) thus limiting the ability of these models to identify strong environmental associations. Or, as we are limited to the resolution of geolocation derived predator data, meaningful habitat-kittiwake associations maybe operating at smaller scales (both spatial and temporal), then we are able to quantify [28, 79]. Regardless, overall our results show that multiple pelagic habitats are suitable for wintering kittiwakes.

In 2009/10, El Niño Modoki conditions occurred (central Pacific El Niño), characterized by a weakened Aleutian Low which is strengthened during a typical El Niño winter [80]. During this winter the western sub-arctic experienced anomalously high sea surface temperatures, while the central subarctic had anomalously cool sea surface temperatures [81]. The distribution of wintering kittiwakes in 2009/10 had a restricted longitudinal range and birds stayed much longer in the Bering Sea, potentially facilitated by colder sea surface temperatures (see Fig. 4 and Table 6), than during 2010/11, classified as strong La Niña year or 2008/09 classified as neutral conditions [82]. This shift to more northerly distributions in 2009/10 was also noted for wintering kittiwakes originating from the Shoup Bay colony in Prince William Sound [33]. This similar response suggests that during 2009/10 conditions suitable for wintering kittiwakes shifted northward across the North Pacific.

**Table 6:** Habitat characteristics of wintering locations for black-legged kittiwakes (*Rissa tridactyla*) from October thru February in 2008/09, 2009/10, and 2010/11

	2008/09	2009/10	2010/11
Bering Sea			
Residency Time (d)	2.1 ± 1.0	2.8 ± 1.0	2.0 ± 0.8
SST (°C)	4.65 ± 2.2	5.23 ± 0.94	5.87 ± 1.85
Distance to Seamount (km)	455 ± 285	242 ± 133	362 ± 230
SSH (cm)	−9.12 ± 3.78	−8.34 ± 2.06	−10.96 ± 3.35
EKE (cm^2^ s^−2^)	70.4 ± 35.9	39.1 ± 15.9	54.6 ± 27.2
Distance to Eddy Edge (km)	143 ± 71	131 ± 48	169 ± 84
Chl a (mg m^−3^)	1.40 ± 0.99	1.68 ± 2.21	2.25 ± 2.79
Wind speed (m s^−1^)	9.17 ± 1.39	8.94 ± 0.70	8.49 ± 1.48
Subarctic Gyre (West)			
Residency Time (d)	2.0 ± 0.7	2.2 ± 0.9	2.0 ± 0.5
SST (°C)	7.75 ± 1.95	5.61 ± 1.78	5.33 ± 1.17
Distance to Seamount (km)	392 ± 130	357 ± 118	348 ± 114
SSH (cm)	−17.99 ± 3.28	−14.83 ± 2.15	−15.49 ± 3.0
EKE (cm^2^ s^−2^)	49.4 ± 19.1	58.9 ± 18.2	59.0 ± 28.4
Distance to Eddy (km)	127 ± 29	124 ± 21	114 ± 11
Chl a (mg m^−3^)	0.63 ± 0.22	0.55 ± 0.13	0.66 ± 0.32
Wind speed (m s^−1^)	8.85 ± 1.52	9.64 ± 1.25	9.46 ± 0.90
Subarctic Gyre (East)			
Residency Time (d)	2.0 ± 0.7	2.1 ± 0.8	1.8 ± 0.7
SST (°C)	7.82 ± 1.34	6.33 ± 1.54	6.58 ± 1.22
Distance to Seamount (km)	766 ± 221	770 ± 228	744 ± 192
SSH (cm)	−14.78 ± 5.39	−10.79 ± 3.48	−13.55 ± 2.89
EKE (cm^2^ s^−2^)	38.6 ± 12.6	58.9 ± 76.0	44.3 ± 34.4
Distance to Eddy (km)	152 ± 35	163 ± 40	147 ± 37
Chl a (mg m^−3^)	0.64 ± 0.28	0.48 ± 0.20	0.50 ± 0.21
Wind speed (m s^−1^)	9.31 ± 1.70	9.49 ± 2.13	9.77 ± 2.07
Polar Front			
Residency Time (d)	2.1 ± 0.6	2.0 ± 0.5	1.9 ± 1.3
SST (°C)	9.67 ± 1.22	8.50 ± 1.43	8.83 ± 1.68
Distance to Seamount (km)	119 ± 390	122 ± 324	131 ± 325
SSH (cm)	2.78 ± 6.72	5.30 ± 7.91	−0.89 ± 4.72
EKE (cm^2^ s^−2^)	75.2 ± 23.8	67.7 ± 18.9	55.3 ± 32.5
Distance to Eddy (km)	119 ± 21	122 ± 22	131 ± 42
Chl a (mg m^−3^)	0.35 ± 0.05	0.32 ± 0.05	0.32 ± 0.06
Wind speed (m s^−1^)	9.76 ± 0.95	9.91 ± 0.84	9.19 ± 2.61

Yearly means ± SD are calculated from individual bird means in each ecoregion

The winter of 2010/11, switched to one of strong La Niña conditions [83]. In the Gulf of Alaska, zooplankton biomass, survival estimates for age-1 pollock, and catch rates of juvenile pink salmon were all low [84, 85]. In 2010/11, kittiwakes spent much less time in the Polar Front region, however birds that did venture there did not experience conditions that were notably different from either of the other years, except for a marked decrease in EKE. Eddies and surface currents are known to condense and facilitate prey capture for surface foraging seabirds [28, 79], thus is may be that this difference is linked to lower use. Relative to 2008/09 the more northerly distributions in 2009/10, when birds used the Bering Sea more, and in 2010/11 when kittiwake range in the subarctic was decreased, could be closer to what kittiwake wintering distributions may be like in the future.

Climate change is predicted to shift the North Pacific Transition Zone farther north, causing the subarctic zone south of the Aleutians to shrink in size [86]. This shift in habitats is likely to be challenging for Hawaiian albatrosses as it moves preferred foraging areas farther from breeding colonies [87, 88]. For kittiwakes, these changes will shrink the area of available wintering habitat, initially to a greater extent than what will open up to the north (due to the presence of land). Furthermore, in the North Pacific, Russian stocks of pink salmon (*Oncorhynchus gorbuscha*) currently follow a predictable alternation between large and small cohorts [89]. During large cohort years, prey availability is suppressed and across the North Pacific black-legged kittiwakes respond through later hatch dates, lower laying success, smaller clutch size, and lower overall reproductive productivity—these effects appear to diminish in colonies in the Gulf of Alaska [89]. A shrinking subarctic may exacerbate competition between pink salmon, other salmonids, and seabirds. This could then lead to density dependent regulation of kittiwake populations, particularly if winter day length limits how far north kittiwakes can remain during the winter [90].

Though black-legged kittiwakes wintering in the pelagic North Pacific are able to retain spatial memories from one year to the next they also appear to have a high capacity to shift distributions in relationship to annual conditions. It remains unknown how long spatial memories may persist and if these long-lived individuals are simply returning to areas visited in the more distant past. It is also unknown if individuals are targeting previously profitable foraging areas. We were unable to find strong associations between kittiwake residency time and oceanographic habitats. This may reflect the scale of our analysis or be a characteristic that highlights the capacity of black-legged kittiwakes to use multiple wintering ecoregions, presumably foraging on different prey species across both space and time.

## Availability of supporting data

Raw geolocation data are available from the North Pacific Research Board [91].
